# Ultra High-Mass Resolution Paper Spray by Fourier Transform Ion Cyclotron Resonance Mass Spectrometry

**DOI:** 10.1155/2012/382021

**Published:** 2012-04-23

**Authors:** Kevin D. Quinn, Charmion I. Cruickshank, Troy D. Wood

**Affiliations:** Department of Chemistry, State University of New York at Buffalo, Buffalo, NY 14260, USA

## Abstract

Paper Spray Ionization is an atmospheric pressure ionization technique that utilizes an offline electro-osmotic flow to generate ions off a paper medium. This technique can be performed on a Bruker SolariX Fourier transform ion cyclotron resonance mass spectrometer by modifying the existing nanospray source. High-resolution paper spray spectra were obtained for both organic and biological samples to demonstrate the benefit of linking the technique with a high-resolution mass analyzer. Error values in the range 0.23 to 2.14 ppm were obtained for calf lung surfactant extract with broadband mass resolving power (m/Δm_50%_) above 60,000 utilizing an external calibration standard.

## 1. Introduction

Since the first practical implementation of electrospray ionization (ESI) with mass spectrometry reported by Yamashita and Fenn in 1984, a number of different atmospheric pressure ionization (API) techniques have been developed [3]. Many API techniques require extensive sample preparation as well as costly consumables to obtain mass spectra. One of the most cost effective and widely available media for chemical separation and analysis is paper. It has been shown that an electro-osmotic flow similar to that of static electrospray can be utilized to interface a triangle of wetted paper to a mass spectrometer (MS); this form of analysis is called paper spray (PS) [4–9].

The use of a chromatographic medium such as paper allows for the analysis of complex mixtures since separations will occur as the analyte is being driven along the paper medium [7]. Separating analytes on a paper medium provides the benefits of reduced probability of ion suppression and rapid analysis. The paper medium allows for analysis of complex samples such as biological tissue and dried blood spots which are not suitable for traditional infusion techniques due to their complex matrices [4, 7].

One limitation to PS, like many MS techniques that utilize incomplete chromatographic separations is the detector being utilized to perform the analysis. When dealing with complex samples or unknown samples, there is a need for high-quality data in order to determine what is being analyzed. Fourier transform ion cyclotron resonance (FT-ICR) mass spectrometers are powerful tools for analysis of complex mixtures due to their unsurpassed capability to obtain broadband high-resolution mass spectra with high mass accuracy. Utilizing PS with a Fourier transform ion cyclotron resonance (FT-ICR) mass spectrometer as a detector allows for rapid analysis of both unknowns and/or complex mixtures.

In this present work, we show the suitability of a 12 Tesla FT-ICR MS to obtain high-resolution broadband mass spectra utilizing a coffee filter paper spray medium. High-resolution mass spectra were first obtained for polypropylene glycol (PPG) and polyethylene glycol (PEG) standards to test the source. Second, urine and calf lung surfactant extract (CLSE) were analyzed to test the PS source for analysis of complex mixtures not suitable for direct infusion.

## 2. Materials, Methods, and Instrumentation

### 2.1. Chemicals

All solvents used were HPLC grade obtained from Sigma Aldrich (St. Louis, MO, USA). PPG was obtained in the form of a tuning standard from Applied Biosystems (Bedford, MA, USA). PEG standard was made by dissolving 1 mg of PEG 2000 obtained from Sigma Aldrich (St. Louis, MO, USA) in 1 mL of HPLC grade water. Coffee filter, Wegmans brand (Rochester, NY, USA), was cut by hand into an isosceles triangle with dimensions of approximately 5 mm (base) by 10 mm (height).

### 2.2. PPG and PEG

Samples for PPG and PEG PS analysis were prepared by wetting the coffee filter triangle with 10 *μ*L of the standard solutions. The wet filter paper samples were allowed to dry under ambient conditions before PS analysis was performed. Mass spectra were then obtained by wetting the dried paper triangle with 10 *μ*L of 50 : 50 methanol : water and using the method described in Section 2.5.

### 2.3. CLSE

CLSE was obtained from ONY Inc. (Amherst, NY, USA). Prior to analysis, the sample was stored in chloroform in the refrigerator. Samples for CLSE analysis were produced by wetting a coffee paper triangle with 2 *μ*L of CLSE and allowing the sample to dry under ambient conditions before PS analysis was performed. Mass spectra were then obtained by wetting the paper with 10 *μ*L of 50 : 50 methanol : water using the instrument method described in Section 2.5.

### 2.4. Urine

Urine samples were collected from two healthy adult males into sterile polypropylene centrifuge tubes (VWR Scientific) and immediately placed in the freezer. The samples were collected in accordance with the Health Sciences Institutional Review Board at the University at Buffalo (HSIRB no. CHE0050411E). Prior to analysis, the samples were thawed to room temperature and 1 mL was pipetted into 2 mL sample vials (Agilent, Santa Clara, CA, USA) for use. Ten *μ*L was injected directly onto a triangular coffee filter and allowed to dry under ambient conditions. Once dry, the filter paper was attached to the mass spectrometer via a copper clip and rewetted using 50 : 50 methanol : water for PS analysis using the instrument method described in Section 2.5.

### 2.5. Paper Spray FT-ICR Method

**Figure 1:**
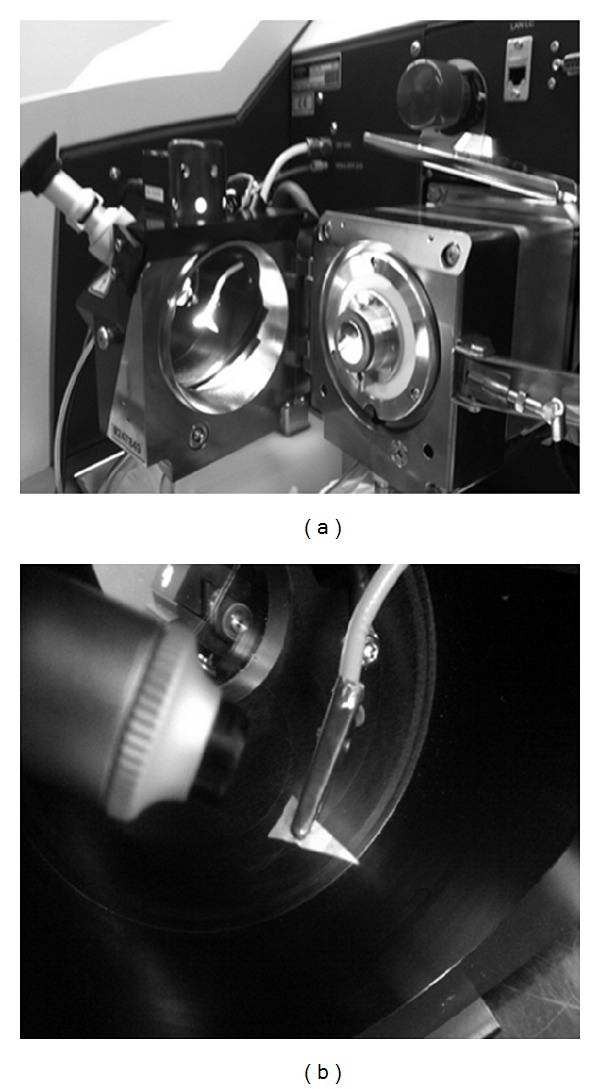
PS source utilizing a modified Bruker nano-ESI source, a copper clip, and cable to ground the paper triangle to the chasse of the instrument.

High-resolution spectra were obtained on a 12T Bruker SolariX FT-ICR MS (Bruker Daltonics Inc., Billerica, MA, USA) utilizing a modified Bruker nanoelectrospray source. The positive potential was obtained by grounding the paper triangle using a copper clip and applying a negative voltage at the inlet to the mass spectrometer (Figure 1 shows an image of the source). Mass spectra were obtained at various mass to charge ranges with a pre-FT-ICR-trap hexapole accumulation time of 0.400 seconds and an FT-ICR trap accumulation time of 0.025 seconds. All mass spectra were obtained in the positive mode at a spray voltage of 2 kV. Mass resolving power (m/∆m_50%_) was defined in accordance with the literature [10].

## 3. Results and Discussion

### 3.1. Polymer Standards

**Figure 2:**
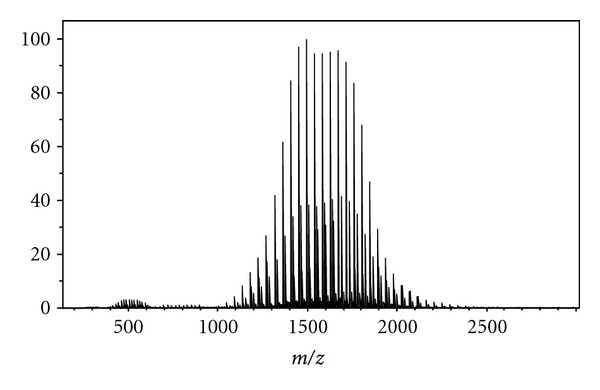
Polyethylene glycol paper spray mass spectrum; identification of oligomers as given in the literature [1].

**Figure 3:**
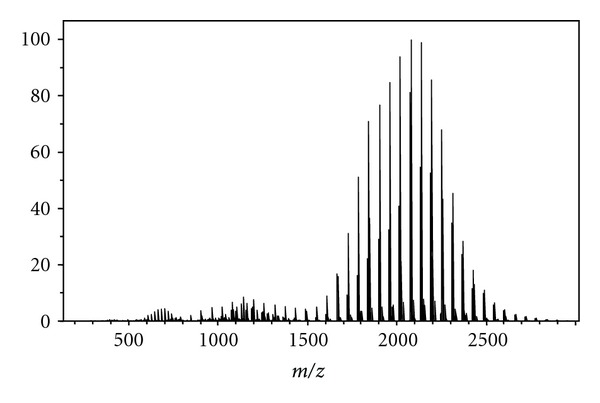
Polypropylene glycol paper spray mass spectrum; identifications of oligomers as given in the literature [2].

 Polymeric standards were utilized to determine the suitability of PS with high-resolution FT-ICR MS. In FT-ICR MS the resolution of the mass spectra is relative to *m/z* as well as the number of data points that can be obtained during the detection process. High-resolution spectra require a time domain signal stable for longer than several hundred milliseconds. Polyethylene glycol and polypropylene glycol produce a broad *m/z* range of ions ideal for testing the suitability of PS with high-resolution mass spectrometry. High-resolution mass spectra of PEG and PPG are displayed in Figures 2 and 3, respectively. Identifies of the oligomer ion detected and their associated errors is given in Tables 1 and 2, respectively.

Signal-to-noise obtained for PEG and PPG after tuning spectra was in excess of 100/1 with absolute intensities that varied shot-to-shot by less than 20%. This allowed ample time for adjustment of the filter paper towards the inlet of the mass spectrometer so as to optimize instrument setup and analysis. Utilizing polymers of known *M*_*n*_ values (provided by the manufacturers; polydispersity not given) such as PEG 2000 and PPG 2000 provides a detection window several hundred *m/z* wide for broad mass accuracy calibrations for FT-ICR MS. 

### 3.2. Calf Lung Surfactant Extract

**Figure 4:**
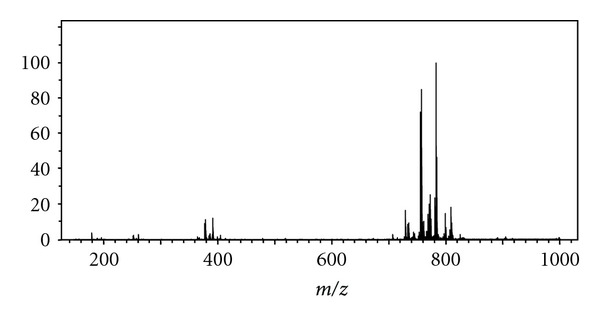
Broadband PS mass spectrum of 2 *μ*L CLSE, the spectrum was obtained by signal averaging 48 scans (arbitrary).

**Figure 5:**
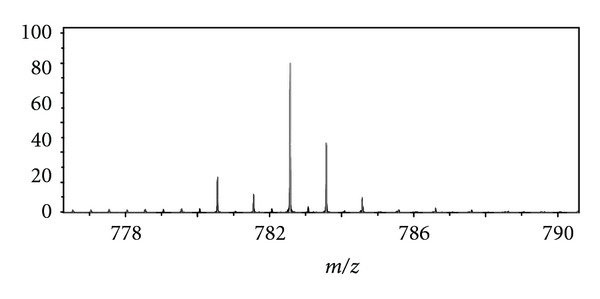
Inset of broadband PS mass spectrum of CLSE over *m/z* 776–791.

 Calf lung surfactant extract (CLSE), which is commercially available in drug form as Infasurf, is used to treat respiratory distress syndrome [11]. CLSE is obtained by doing a total lipid extraction on the lavage fluid from a slaughtered calf lung [11, 12]. Phosphatidylcholine (PC) makes 79% of the molar distribution of lipids and consists of a choline head group attached to the phospholipid with varying degrees of unsaturation in the fatty acid chains [2]. Based on the optimized setup determined from previously analyzing the standards, CLSE was then analyzed. PS mass spectra of CLSE are given in Figures 4 and 5, and the identities with associated error provided in Table 3. 

### 3.3. Urine

**Figure 6:**
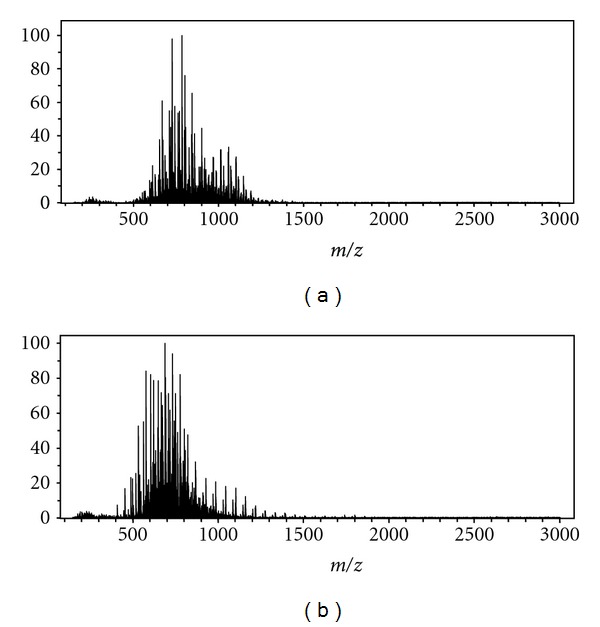
PS mass spectra of urine specimens from two healthy male individuals: 27 year-old (a) and 44 year-old (b).

**Figure 7:**
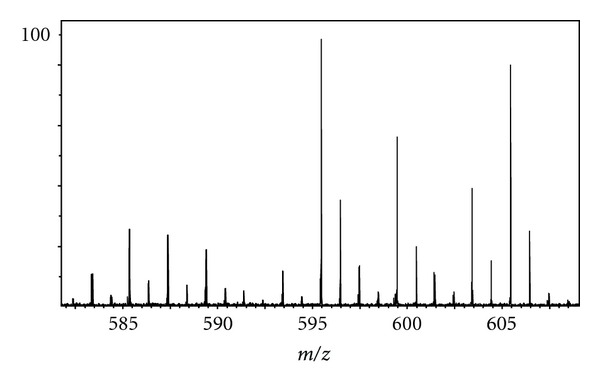
Inset of PS mass spectrum for urine specimen from 27-year-old volunteer over *m/z* 582–608.

**Figure 8:**
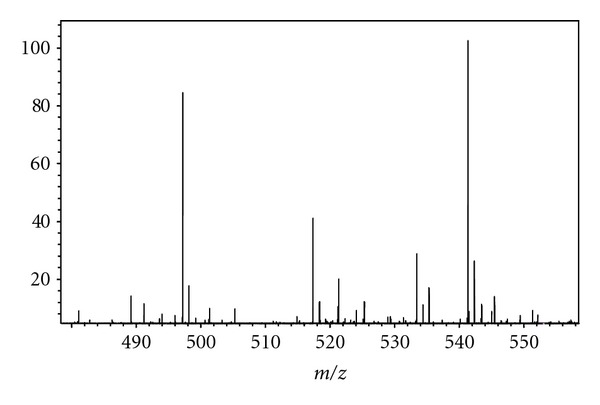
Inset of PS mass spectrum for urine specimen from 44-year-old volunteer being treated with prescription vitamin D supplement over *m/z* 480–560.

Due to the complex nature of urine, many small molecules and metabolites were detected at varying intensities using PS with FT-ICR. PS mass spectra, showing numerous lipids and other metabolites obtained from the analyzed urine specimens of two healthy adult male subjects, are shown in Figure 6; an inset of one of these PS mass spectra is shown in Figure 7 while the other is shown in Figure 8. A few of the metabolites identified in the 27-year-old volunteer using the exact mass capability of FT-ICR are provided in Table 4. Metabolites identified in the 44-year-old volunteer using the exact mass capability of FT-ICR are given in Table 5. Of note, the 44-year-old volunteer, who was taking prescribed vitamin D supplements, showed indications of the vitamin D metabolite 25-Hydroxyvitamin D2-25-glucuronide in the urine. Interestingly, both adult male subjects had indications of one or more glucuronides in urine. This may signal a particularly strong propensity for glucuronides to ionize via the PS ionization mechanism; this capability of PS should be further explored. 

## 4. Conclusions

We developed a PS source for Bruker FT-ICR instrumentation that can be utilized for a broad range of chemical species. The PS source was tested on a range of samples and determined the source was suitable for both polymer and biological samples. The PS source provided an API medium for the analysis of complex mixtures while mitigating the possibility of cross contamination due to the disposable nature of coffee filter paper.

## Figures and Tables

**Table 1:** PEG standards identified using paper spray mass spectrometry. The PEG mass spectrum was obtained with both an excitation and detection frequency range of 1.5 MHz.

PEG	Experimental *m/z *	Theoretical *m/z *	Mass measurement error (Δppm)	Mass resolving power (M/ΔM_50%_)	[H(OCH_2_CH_2_)*_n_*OH+Na]^+^
	1009.577639	1009.57651	−0.12	46309	*n* = 22
	1053.60274	1053.60273	0.01	42870	*n* = 23
	1097.62886	1097.62894	−0.07	40306	*n* = 24
	1141.65527	1141.65515	0.02	38791	*n* = 25
	1185.68128	1185.68137	−0.08	37199	*n* = 26
	1229.70779	1229.70758	0.17	35953	*n* = 27
	1273.73391	1273.7338	0.09	34776	*n* = 28
	1317.76035	1317.76001	0.26	33645	*n* = 29
	1361.78640	1361.78623	0.12	32579	*n* = 30
	1405.81232	1405.81244	−0.09	31288	*n* = 31
	1449.83868	1449.83866	0.01	30101	*n* = 32
	1493.86539	1493.86487	0.35	29219	*n* = 33
	1537.89172	1537.89109	0.41	28219	*n* = 34
	1581.91742	1581.9173	0.08	27254	*n* = 35
	1625.94358	1625.94352	0.04	26391	*n* = 36
	1669.96968	1669.96973	−0.03	25567	*n* = 37
	1713.99520	1713.99595	−0.44	24691	*n* = 38

**Table 2:** PPG standards identified using paper spray mass spectrometry. The PPG mass spectrum was obtained with excitation and detection frequency range of 1.5 MHz.

PPG	Experimental *m/z *	Theoretical *m/z *	Mass measurement error (Δppm)	Mass resolving power (M/ΔM_50%_)	[C_3_O_2_H_7_(C_3_H_6_O)*_n_*C_6_H_13_O_2_+Na]^+^
	1666.17197	1666.17200	−0.02	22686	*n* = 25
	1724.21224	1724.21386	−0.94	23629	*n* = 26
	1782.25781	1782.25573	1.17	23817	*n* = 27
	1840.29829	1840.29760	0.37	23808	*n* = 28
	1898.34113	1898.33946	0.88	23139	*n* = 29
	1956.37992	1956.38133	−0.72	22968	*n* = 30
	2014.41937	2014.42319	−1.90	22638	*n* = 31
	2072.46849	2072.46505	1.66	21991	*n* = 32
	2130.50088	2130.50692	−2.84	21503	*n* = 33
	2188.55370	2188.54878	2.25	20679	*n* = 34
	2246.59219	2246.59065	0.69	19685	*n* = 35
	2304.63182	2304.63254	−0.31	17868	*n* = 36
	2362.67370	2362.67438	−0.29	16365	*n* = 37

**Table 3:** CLSE compounds identified using paper spray mass spectrometry. The phospholipids detected are denoted using the lipid numbers nomenclature to indicate degrees of unsaturation, and PC refers to phosphatidylcholine. The CLSE mass spectrum was obtained with excitation and detection frequency range of 1.5 MHz.

Experimental *m/z *	Theoretical *m/z *	Mass measurement error (Δppm)	Mass resolving power (M/ΔM_50%_)	Compound
728.52151	728.52008	1.96	61637	16 : 0/14 : 0 PC [M+Na]^+^
732.55505	732.55378	1.73	60967	16/16 PC [M+H]^+^ 1 degree of unsaturation
734.57100	734.56943	2.14	62820	16 : 0/16 : 0 PC [M+H]^+^
754.53660	754.53572	1.17	57074	16/16 PC [M+Na]^+^ 1 degree of unsaturation
756.55257	756.55137	1.59	57597	16 : 0/16 : 0 PC [M+Na]^+^
760.58591	760.58508	1.09	55950	16/18 PC [M+H]^+^ 1 degree of unsaturation
782.56728	782.56702	0.33	52802	16/18 PC [M+Na]^+^ 1 degree of unsaturation
798.54114	798.54096	0.23	48188	16/18 PC [M+K]^+^ 1 degree of unsaturation
808.58322	808.58267	0.68	48764	18/18 PC [M+Na]^+^ 2 degrees of unsaturation

**Table 4:** Compounds identified using paper spray mass spectrometry on urine sample from 27-year-old volunteer. The urine mass spectrum was obtained with both an excitation and detection frequency range of 1.5 MHz.

Compound	Species	Formula	Experimental *m/z *	Theoretical *m/z *	Mass measurement error (Δppm)
3-Hexaprenyl-4,5-Dihydroxybenzoic acid	[M + Na]	C_37_H_54_O_4_Na	585.39288	585.39143	2.5
3′-hydroxy-e,e-caroten-3-one	[M + CH_3_OH + H]	C_41_H_59_O_3_	599.44790	599.44587	3.4
Vitamin D2 3-glucuronide	[M + CH_3_OH + H]	C_35_H_57_O_8_	605.40328	605.40480	2.5
Coenzyme Q10	[M + K]	C_59_H_90_O_4_K	901.64549	901.64707	1.8

**Table 5:** Compounds identified using paper spray mass spectrometry on urine sample from 44-year-old volunteer.  The urine mass spectrum was obtained with excitation and detection frequency range of 1.5 MHz.

Compound	Species	Formula	Experimental *m/z *	Theoretical *m/z *	Mass measurement error (Δppm)
Vitamin K1	[M + K]	C_31_H_46_O_2_K	489.31176	489.31294	2.4
16-alpha, 17-beta-estriol 17-beta-D-glucuronide	[M + CH_3_OH + H]	C_25_H_37_O_10_	497.23617	497.23812	3.9
Tetrahydroaldosterone-3-glucuronide	[M + H]	C_27_H_41_O_11_	541.26279	541.26434	2.9
25-Hydroxyvitamin D2-25-glucuronide	[M + CH_3_OH + H]	C_35_H_57_O_9_	621.39946	621.39971	0.4

## Nomenclature

ESI: Electrospray ionization
API: Atmospheric pressure ionization
MS: Mass spectrometer
PS: Paper spray
FT-ICR: Fourier transform ion cyclotron resonance
PPG: Polypropylene glycol
PEG: Polyethylene glycol
CLSE: Calf lung surfactant extract
PC: Phosphatidylcholine
HSIRB: Health Sciences Institutional Review Board.
